# Supplementary material for: Spexin and a Novel Cichlid-Specific Spexin Paralog Both Inhibit FSH and LH Through a Specific Galanin Receptor (Galr2b) in Tilapia
Source: Front Endocrinol (Lausanne). 2020 Feb 20;11:71. doi: 10.3389/fendo.2020.00071 (PMC7044129; doi:10.3389/fendo.2020.00071)
Supplement: Supplementary file 1 [file Data_Sheet_1.PDF]

Supplementary

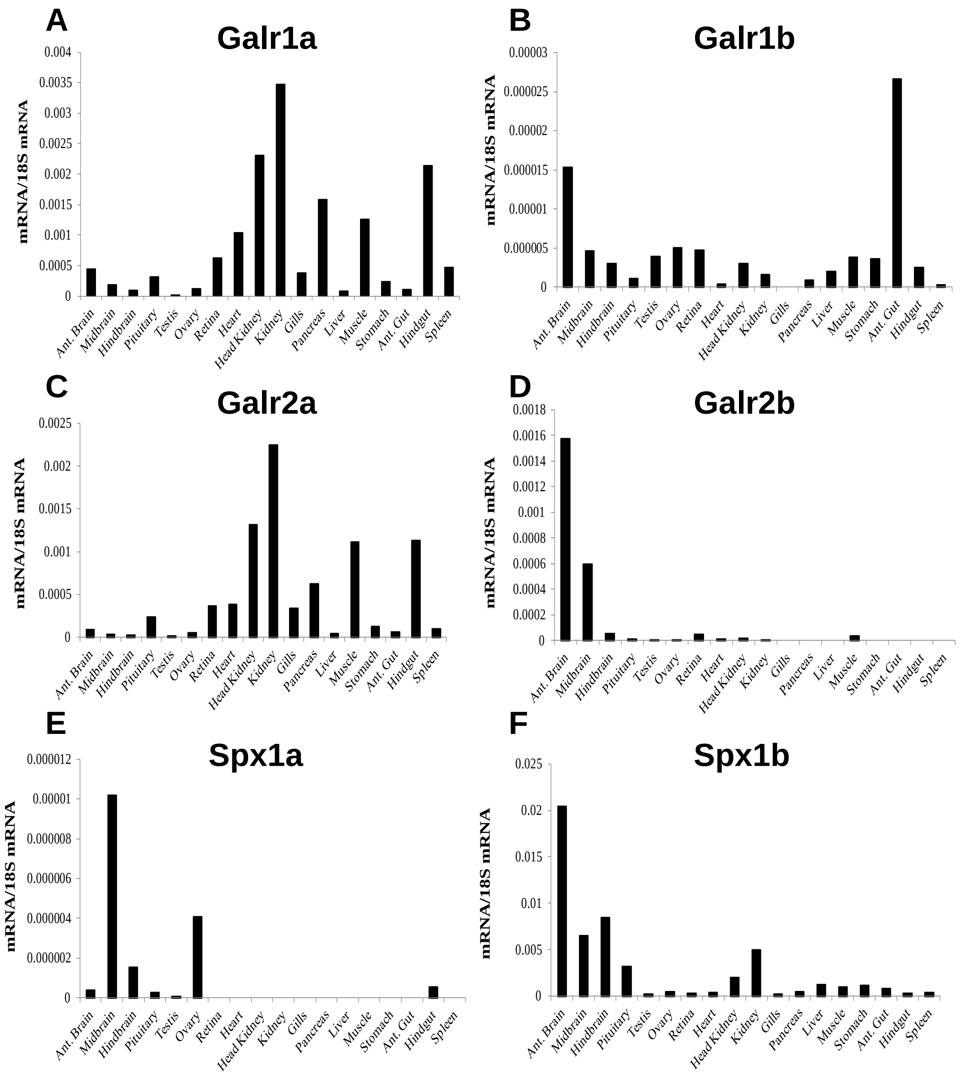

Supplementary Figure 1. Tissue distribution of tilapia SPXs and GALRs. The genes were normalized against an endogenous reference (18S) by the comparative cycle threshold method.

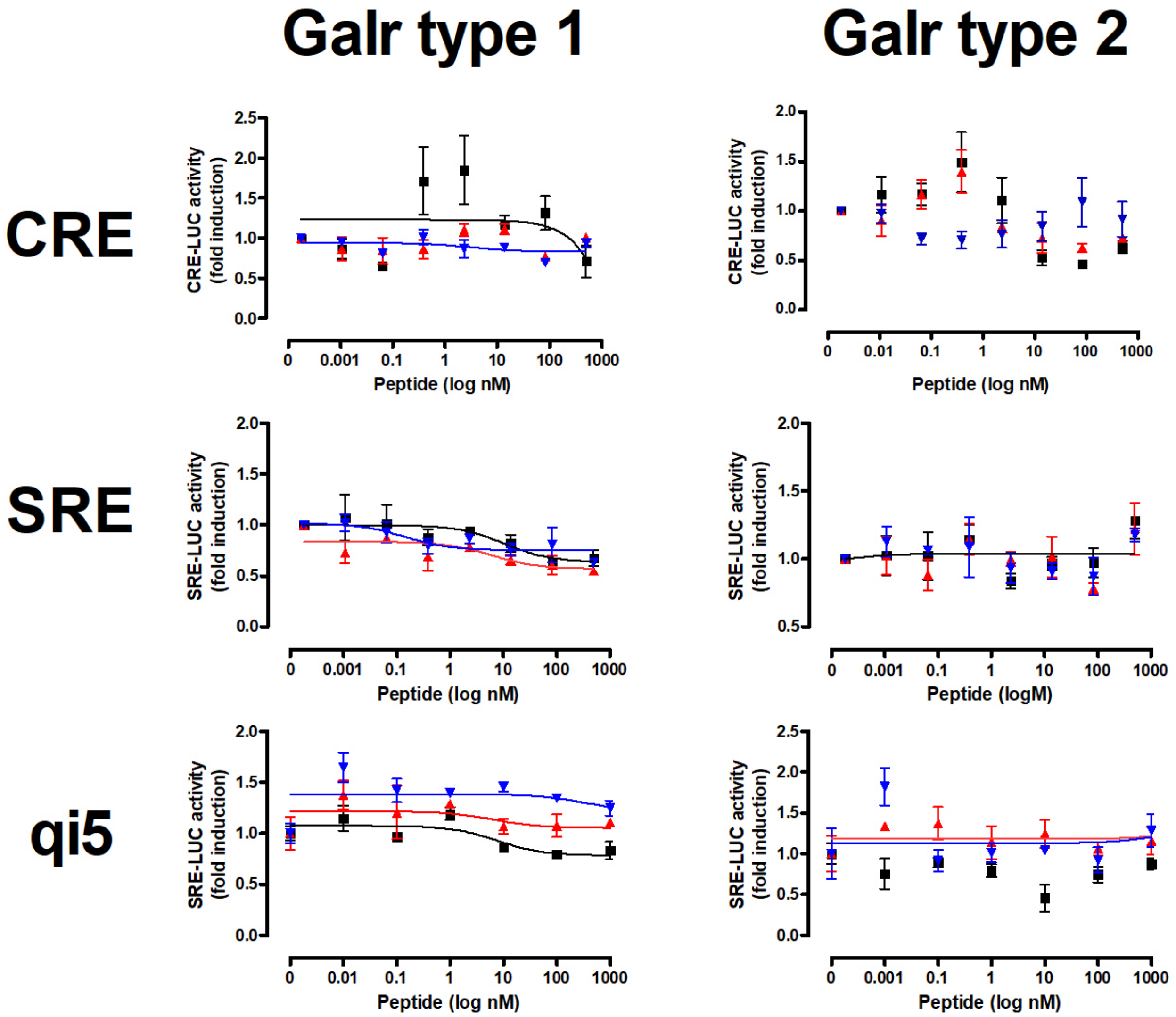

Supplementary Figure 2. In vitro reporter assays for tilapia Galanin receptors. Tilapia Galr type 1 or type 2 were cotransfected with either CRE-luc , SRE-Luc, or Gqi5 into Cos7 cells and treated with either Spx1a (black squares), Spx1b (red triangles), or human galanin (Gal; blue upside-down triangles). Data are represented as mean ± SEM fold change in luciferase activity over basal (no treatment).

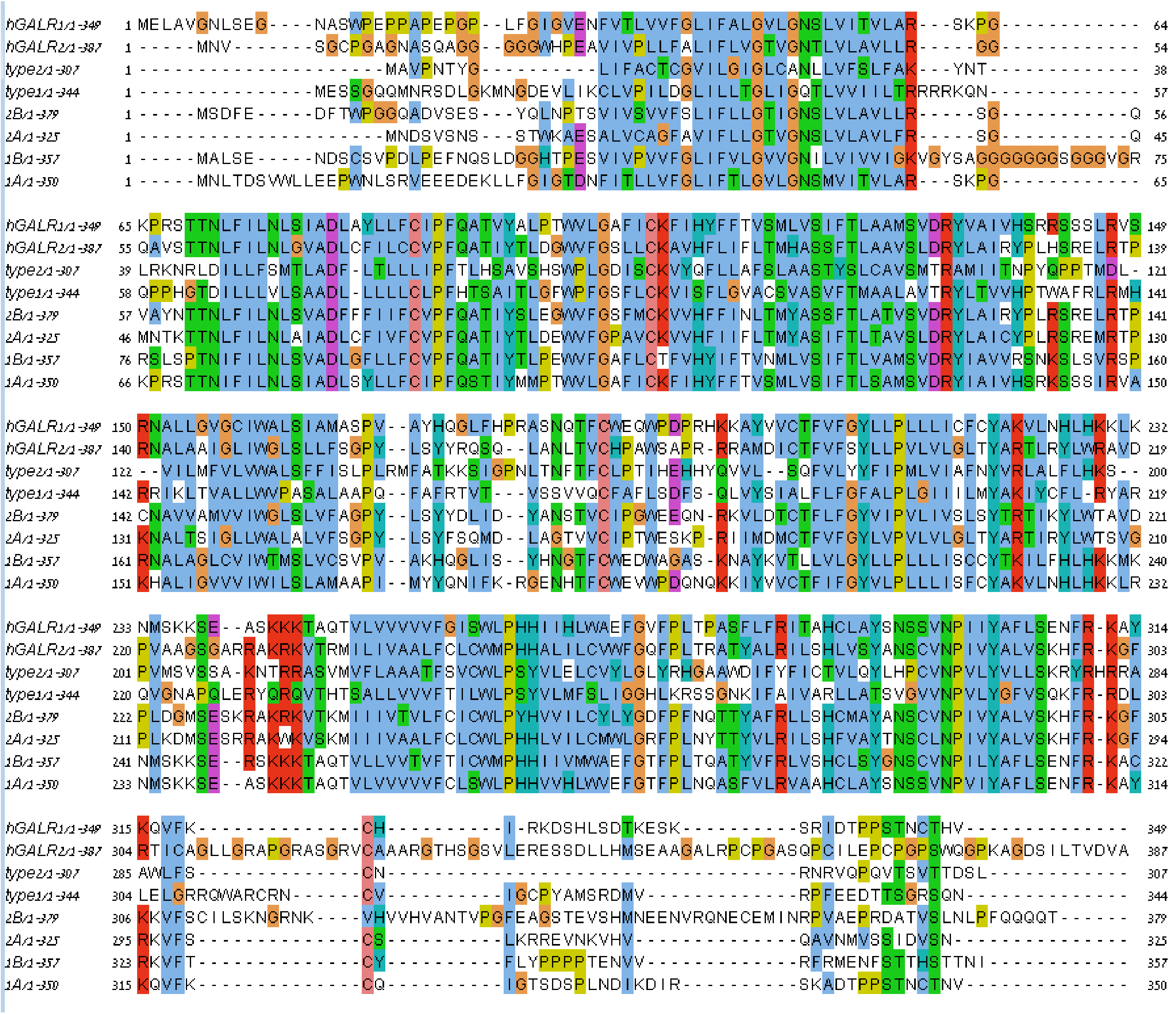

Supplementary Figure 3. Multiple sequence alignment of Galanin receptors. hGALR1, hGALR2, Galr1a, Galr1b, Galr2a, Galr2b, Galr type 1, and Galr type2 amino acid sequences were aligned by MUSCLE, visualized in Jalview, and colored according to ClustalW conservation.

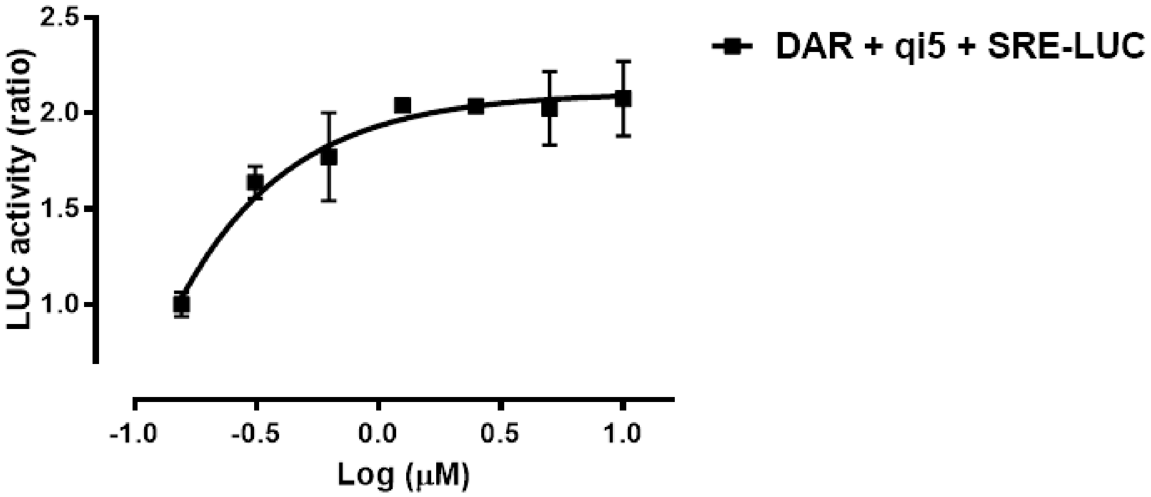

EC50	~ 0.006253

Supplementary Figure 4. ***In vitro*** **reporter assays for tilapia D2 dopamine receptor.** Tilapia D2 dopamine receptor was cotransfected with SRE-Luc and Gqi5 into Cos7 cells and treated with dopamine. Gqi5 allows inhibitory effects to be visualized as stimulus. Data are represented as mean ± SEM fold-change in luciferase activity over basal (no treatment).

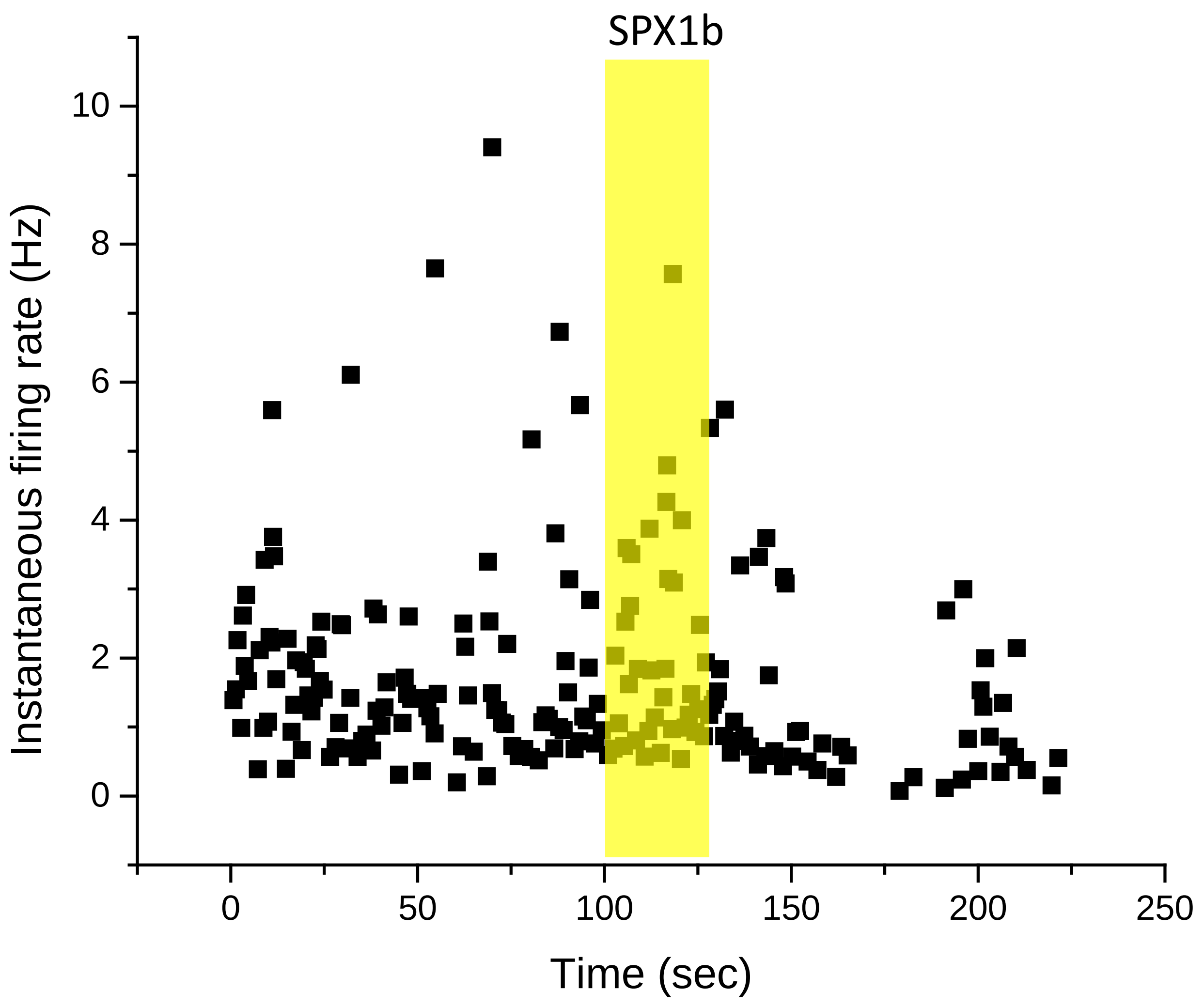

Sopplementary Figure 5. Effect of spexin 1b on firing rate of LH cells in mature tilapia. The pituitary slice was briefly exposed to Spx1b during the recording (boxed). Graphic representation of firing-rate before and during Spx1b application. Each dot represents an action potential along the time scale (X-axis) and the instantaneous frequency from the action potential before (Y-axis).
